# Microbially-induced carbonate precipitation in coal-associated environments: opportunities and challenges

**DOI:** 10.3389/fmicb.2026.1769675

**Published:** 2026-02-24

**Authors:** Kuanysh Tastambek, Azhar Malik, Nuraly Akimbekov, Ilya Digel, Nazym Altynbay, Damir Nussipov, Bekzat Kamenov, Dinara Sherelkhan, Moldir Turaliyeva, Yaya Wang, Xiangrong Liu

**Affiliations:** 1 Sustainability of Ecology and Bioresources, Al-Farabi Kazakh National University, Almaty, Kazakhstan; 2 International Center for Islamic Science and Innovation, Al-Farabi Kazakh National University, Almaty, Kazakhstan; 3 Ecology Research Institute, Khoja Akhmet Yassawi International Kazakh-Turkish University, Turkistan, Kazakhstan; 4 Institute for Bioengineering (IFB) at FH Aachen University of Applied Sciences, Julich, Germany; 5 Department of Biotechnology, M. Auezov South Kazakhstan University, Shymkent, Kazakhstan; 6 College of Chemistry and Chemical Engineering, Xi’an University of Science and Technology, Xi’an, China

**Keywords:** biocementation, dust suppression, microbial-induced carbonate precipitation, urease, ureolytic bacteria

## Abstract

Microbial-induced calcium carbonate precipitation (MICP) has emerged as a promising biotechnological approach for addressing coal dust pollution in mining and industrial environments. Among the various biological agents, urease-producing bacteria play a central role in catalyzing urea hydrolysis, leading to the generation of carbonate ions that react with calcium to form calcium carbonate (CaCO_3_). This biologically formed mineral binds dust particles, enhances surface stability, and reduces airborne pollutant dispersion. While MICP presents clear environmental and structural advantages, including low toxicity, long-term ecological compatibility, and compatibility with natural ecosystems, the underlying mechanisms, particularly the microbial adhesion to coal particles and subsequent mineralization dynamics, remain poorly understood. High production costs, sensitivity to environmental conditions, and lack of large-scale validation have also limited the practical implementation of microbial dust suppressants. This review provides a comprehensive look at the current research on the biological processes and application strategies of MICP in coal dust suppression, emphasizing the role of ureolytic bacteria, carrier systems, and calcium sources. Furthermore, it explores recent advancements in microbial strain selection, additive incorporation, and delivery methods that aim to optimize microbial survival and mineralization efficiency in real-world mining conditions. Future perspectives are discussed to support the development of cost-effective and scalable microbial formulations, paving the way for green and durable solutions in mine dust management.

## Graphical abstract

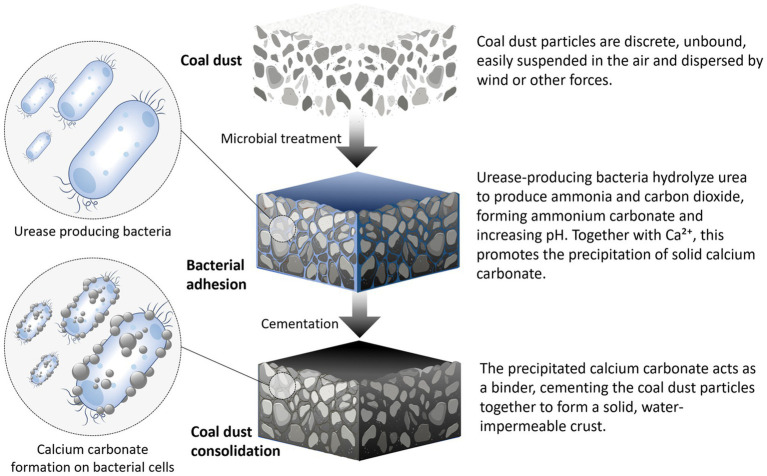

## 1 Introduction

Microbially-induced calcium carbonate precipitation (MICP) is a bio-mediated process in which specific microorganisms trigger the *in situ* precipitation of calcium carbonate (CaCO_3_) through their metabolic activities. It has attracted increasing attention as a sustainable and environmentally friendly alternative to conventional physicochemical methods for site remediation (Seifan et al., 2020). Unlike chemical approaches that are energy-intensive and often generate secondary pollutants, MICP functions under mild conditions, harnessing natural microbial pathways to achieve stabilization, consolidation, or detoxification.

The biological basis of MICP lies mainly in ureolytic bacteria such as *Sporosarcina pasteurii*, *Bacillus mucilaginosus*, *Bacillus sphaericus*, *Paenibacillus mucilaginosus*, *Bacillus megaterium*, and *Staphylococcus succinus* (Zúñiga-Barra et al., 2023). These microorganisms hydrolyze urea into ammonium (NH_4_^+^) and carbonate ions (CO_3_^2−^), which then combine with calcium ions (Ca^2+^) to form CaCO_3_ precipitates, creating consolidated mineral matrices. This reaction mechanism offers several functional benefits, including soil stabilization, permeability reduction, immobilization of heavy metals, neutralization of acid mine drainage (AMD), and long-term carbon sequestration (Zhu et al., 2019; Zhang et al., 2019; Ramachandran et al., 2020; Achal et al., 2016; Achal and Mukherjee, 2015; Yaqoob et al., 2021).

Environmental conditions strongly influence this process. Parameters such as pH, calcium availability, enzyme activity, and microbial viability are critical for efficient carbonate precipitation (Gupta et al., 2017; Muhammad et al., 2016; Aziz et al., 2019; Vijay et al., 2017; Al-Salloum et al., 2017; Mugwar and Harbottle, 2016; Yaqoob et al., 2020; Wong, 2015). Additionally, extracellular polymeric substances (EPS) secreted by microbes enhance ion binding and promote the formation of stable mineral phases (Kang and So, 2016). The morphology of the resulting CaCO_3_ (e.g., calcite, vaterite, or aragonite) depends on both bacterial strain and environmental conditions, ultimately determining the stability and durability of the precipitates (Li et al., 2018).

In the coal industry, MICP holds particular significance due to its potential for controlling coal dust. Open-pit mining, while economically important, generates high volumes of dust during drilling, blasting, excavation, and transportation (Wang Z. et al., 2024; Yang et al., 2024). This dust not only poses occupational hazards, contributing to diseases such as pneumoconiosis, but also is an environmental pollutant capable of long-range dispersion (Sairanen et al., 2018; Xu et al., 2023; Dong et al., 2024). Furthermore, abrasive coal dust accelerates equipment wear and raises the risk of explosions (Zhou et al., 2023a). These problems highlight the urgent need for dust suppression strategies that are both effective and environmentally sustainable (Kamran et al., 2024).

Traditional methods such as water spraying and chemical dust suppressants suffer from limitations, including high evaporation rates, water scarcity, and ecological risks due to toxicity and poor biodegradability (Li L. et al., 2021). In contrast, MICP offers a low-cost, biodegradable alternative by generating natural CaCO_3_ crusts that bind fine particles and provide long-lasting dust suppression (Bao et al., 2020; Zhou et al., 2023b). Moreover, the use of indigenous or engineered bacteria adapted to coal mining environments enhances feasibility and reduces treatment costs.

This review provides a comprehensive overview of recent advances in microbial technologies used in MICP for environmental applications, with a particular emphasis on coal dust management. By examining microbial behavior and comparing the outcomes of various MICP studies, we aim to identify promising bacterial strains for targeted applications. These insights will likely facilitate future research endeavors aimed at optimizing MICP processes for practical and scalable use. The review also highlights the broader relevance of MICP in environmental remediation, with a specific focus on its applicability to the coal industry and its potential to foster sustainable carbonate precipitation strategies.

## 2 Biology of microbial-induced calcium carbonate precipitation

### 2.1 Mechanisms of MICP

#### 2.1.1 Urease-mediated pathway

**Figure 1:**
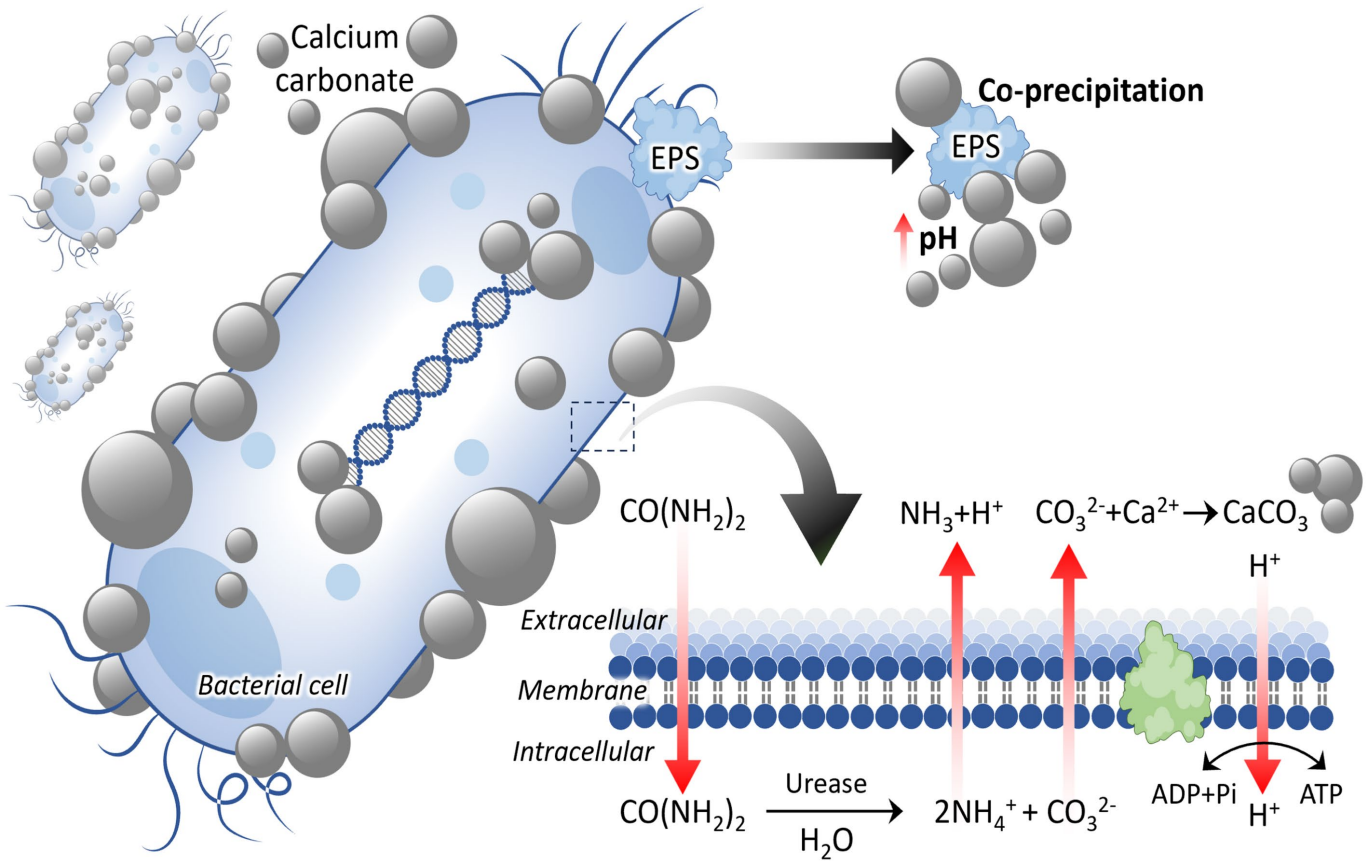
Microbial-induced carbonate precipitation mechanism. EPS, extracellular polymeric substances.

MICP is driven by specific bacterial activity, primarily through urease or other metabolic pathways (Wu et al., 2020). The fundamental principle of the process is that bacteria hydrolyze urea into NH_4_^+^ and CO_3_^2−^, and these carbonate ions subsequently react with Ca^2+2+^ to form insoluble CaCO_3_ (Figure 1). In addition to enzymatic hydrolysis, microorganisms promote mineral formation by creating a chemically favorable microenvironment around their cells. Their surface biomolecules and extracellular biopolymers possess functional groups capable of coordinating Ca^2+^, thereby stabilizing ion accumulation and promoting subsequent nucleation and growth of carbonate minerals.

**Figure 2:**
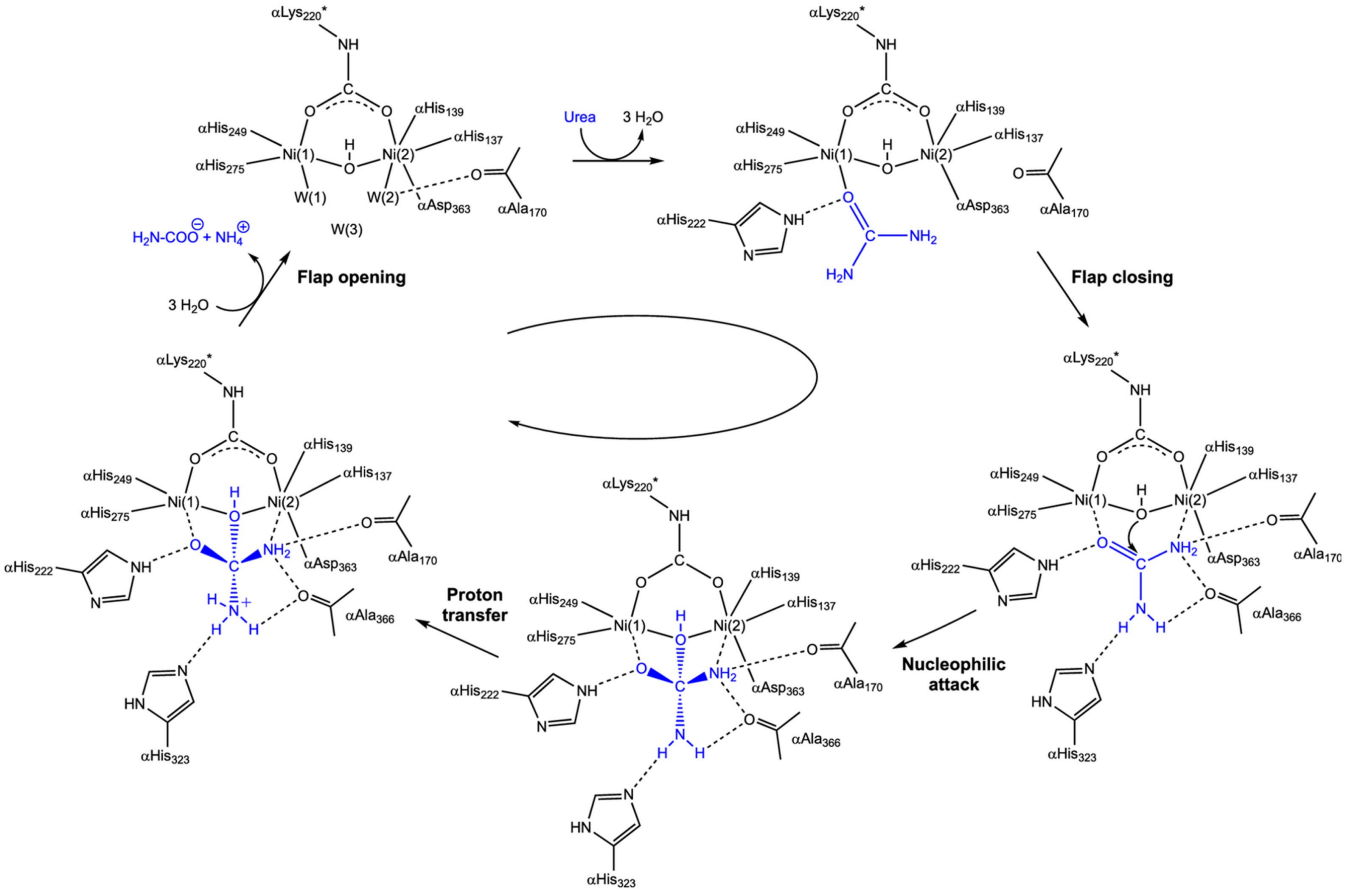
Catalytic mechanism of urea hydrolysis by urease (Mazzei et al., 2020).

Given that these microbially mediated processes ultimately depend on the enzymatic breakdown of urea, a closer examination of urease as the central biocatalyst is essential for understanding the molecular basis of MICP. Urease is a metalloenzyme, specifically a Ni-containing metalloprotein (Gowthaman et al., 2020). Amino acid residues in the active site and those in the flexible flap region are crucial for urea hydrolysis. The flap region of urease regulates the binding of urea and the release of urease, thereby contributing to urea hydrolysis (Jiang, 2021). When the flap region is open, urea enters the active site (Figure 2). The oxygen atom of the carbonyl group is bonded to Ni (1), while the -NH_2_ coordinates with Ni (2), triggering closure of the flap. The proton (H^+^) produced by the -OH group coordinated with the two Ni atoms is transferred to another -NH_2_ group of urea. This protonation activates carbonyl carbon, producing NH_3_ and CO_2_, which are subsequently released. The flap region then reopens. Finally, NH_3_ and CO_2_ react with H_2_O to produce NH_4_^+^ and CO_3_^2−^ (Wu et al., 2020).

The overall chemical reactions can be summarized as follows: Equations (1) and (2):


$$\mathrm{CO}{\left(NH_{2}\right)}_{2}+2H_{2}O\;\overset{\text{urease}}{\to }\;2N{H_{4}}^{+}+C{O_{3}}^{2-}$$ (1)

In the presence of Ca^2+^, these carbonate ions precipitate as CaCO_3_:


$$Ca^{2+}+\text{Cell}\to \text{Cell}-Ca^{2+}$$ (2)

$$\text{Cell}-Ca^{2+}+C{O_{3}}^{2-}\to \text{Cell}-\mathrm{CaC}O_{3}↓$$

The precipitated CaCO_3_ can fill cracks, bind loose particles, and enhance the mechanical integrity of construction materials.

Thus, carbonate ions formed as a result of urease-catalyzed ureolysis are the main biochemical prerequisite for the microbiological precipitation of calcium carbonate, which in turn highlights the importance of considering the role of various microorganisms capable of MICP.

A wide range of microbial species possess the ability to induce MICP, though their efficiency depends largely on environmental parameters and the availability of metabolic substrates. Table 1 provides an overview of 12 well-characterized MICP-active microorganisms, detailing their principal metabolic pathways, biocatalytic processes, and corresponding biotechnological applications. Among them, *S. pasteurii, B. megaterium,* and *S. succinus* primarily employ a ureolytic pathway, whereas *B. sphaericus*, *Pseudomonas* spp., and *Paracoccus* spp. enhances biomineralization efficiency through the combined action of ureolysis and nitrate reduction. In addition to these metabolic routes, several bacterial taxa synthesize extracellular polymeric substances (EPS) that contribute to particle aggregation and matrix cohesion (Shi et al., 2021). For example, *Paenibacillus mucilaginosus* produces EPS capable of reinforcing particle binding and improving soil structural integrity (Zhan et al., 2016). Besides the most common MICP pathways indicated, additional pathways exist that utilize sulfate reductase, methyl coenzyme M reductase, dehydrogenases, oxidases, and carbonic anhydrase (CA), depending on the bacterial groups involved, such as sulfate-reducing bacteria, methanogenic archaea, alkaliphilic aerobic bacteria, and photosynthetic microorganisms. A comprehensive understanding of the metabolic diversity underlying MICP is therefore crucial for the rational selection of bacterial strains tailored to specific environmental or engineering applications.

**Table 1:** The mechanisms by which microbial species evolved in MICP.

MICP pathway/strategy	Key enzymes	Reaction mechanism	Application area	References
Ureolysis-based CaCO_3_ precipitation	Urease	Urea → NH₄^+^ + CO_3_^2−^ → CO_3_^2−^ + Ca^2+^ → CaCO_3_ ↓	Soil stabilization, coal dust control	Li et al. (2024), Khaleghi and Rowshanzamir (2019)
Bio-cementation via ureolysis with additives	Urease	Ureolysis promotes localized CaCO_3_ formation, enhanced by PVA binder	Dust binding on coal surfaces	Du et al. (2024)
Aerobic ureolysis and nitrate reduction	Urease, nitrate reductase	Converts urea and nitrate into carbonate precursors → CaCO_3_ precipitation	Concrete self-healing, mine site biocementation	Wang et al. (2012)
Ureolytic biomineralization + EPS secretion	Urease + EPS	EPS assists particle binding; carbonate from urease-driven hydrolysis binds Ca^2+^ → CaCO_3_	Coal dust suppression, soil binding	Zhu et al. (2016)
Alkaliphilic ureolysis in high pH soils	Urease	Tolerant to alkaline pH; catalyzes urea hydrolysis → carbonate → CaCO_3_	Biocementation in alkaline environments	Sharma et al. (2017)
Ureolytic precipitation under coal conditions	Urease	MICP under microaerophilic conditions → CaCO_3_ crusts form on dust particles	Coal dust biocontrol	Song et al. (2019)
Denitrification-driven alkalinization	Nitrate reductase, nitrite reductase	NO_3_^−^ → N_2_ + OH^−^; increase in pH promotes carbonate precipitation with Ca^2+^	Groundwater remediation, denitrification-assisted biocementation	Lin et al. (2021)
Sulfate reduction and alkalinity generation	Sulfate reductase	SO_4_^2−^ → H_2_S + OH^−^; local alkalinity induces CaCO_3_ precipitation	Anaerobic sediments, mine drainage treatment	Jain et al. (2021)
Methanogenesis-coupled carbonate precipitation	Methyl-coenzyme M reductase	CO_2_ + 4H_2_ → CH_4_ + H_2_O; CO_2_ consumption shifts the equilibrium toward carbonate mineralization	Anaerobic digesters, sediments, CO_2_ sequestration	Su and Yang (2021)
Aerobic respiration and alkalinization	Dehydrogenases, oxidases	Organic substrate oxidation → OH^−^ release; Ca^2+^ binding with CO_3_^2−^	Biocementation in alkaline soils	Lian et al. (2006)
Oxygenic photosynthesis-induced carbonate precipitation	CA, RuBisCO	CO_2_ uptake during photosynthesis increases pH → carbonate oversaturation → CaCO_3_ deposition	Microbial mats, bio-concrete and bioremediation of wastewaters	Anbu et al. (2016)
CA-mediated carbonate precipitation	CA	CO_2_ + H_2_O ⇌ HCO_3_^−^ + H^+^; HCO_3_^−^ combines with Ca^2+^ → CaCO_3_ ↓	Dust stabilization and bio-consolidation in coal-associated environments	Vashisht et al. (2018)

PVA, polyvinyl acetate; EPS, extracellular polymeric substances; CA, carbonic anhydrase.

#### 2.1.2 Carbonic anhydrase-mediated (alternative) pathway

In addition to the well-documented ureolytic pathway, non-photosynthetic microorganisms can induce calcium carbonate precipitation through the activity of CA. This zinc-dependent enzyme catalyzes the reversible hydration of carbon dioxide into bicarbonate ions (CO_2_ + H_2_O ⇌ HCO_3_^−^ + H^+^), thereby increasing the local carbonate concentration available for reaction with Ca^2+^ ions. The CA pathway does not require urea hydrolysis and can function efficiently under conditions where urease activity is limited, making it particularly relevant in coal-associated environments characterized by fluctuating nutrient availability. Several heterotrophic bacteria, including *Pseudomonas* spp., *Bacillus subtilis*, and *Desulfovibrio* spp., have been reported to facilitate calcification via CA activity. Recognition of this mechanism broadens the understanding of microbial versatility in CaCO_3_ precipitation and highlights new opportunities for coal dust suppression strategies where urea supplementation may not be feasible (Du et al., 2024).

Microbially induced calcium carbonate precipitation in coal-associated environments results in the formation of CaCO_3_ polymorphs, primarily vaterite, aragonite, and calcite. The precipitation process typically progresses through a series of transitional phases, beginning with metastable forms (vaterite and aragonite) and culminating in the stable phase (calcite). This mineralogical evolution is strongly influenced by microbial metabolism, physicochemical conditions, and the surface properties of coal dust particles that act as nucleation sites.

1. Initial stage—vaterite formation. Vaterite is often the first polymorph to appear during MICP due to its rapid nucleation kinetics under supersaturated conditions created by ureolysis or carbonic anhydrase activity. In coal dust environments, the fine particulate nature of anthracite or bituminous coal provides abundant nucleation sites that facilitate spherical vaterite crystallization. Although vaterite is thermodynamically unstable, its transient presence is important for initiating particle binding and pore filling in coal dust matrices.
2. Intermediate stage—aragonite deposition. With continued microbial activity, vaterite may transform into aragonite, which commonly crystallizes as needle-like or columnar structures. In coal dust control, aragonite contributes to the mechanical interlocking of particles, enhancing the structural cohesion of dust aggregates. High ionic strength and localized microenvironments created by microbial colonies (e.g., *B. megaterium, S. pasteurii*) favor aragonite stabilization, though it remains less durable compared to calcite (Kim et al., 2016).
3. Final stage—calcite stabilization. Calcite, typically rhombohedral in morphology, emerges as the most stable polymorph under ambient conditions. Over time, metastable vaterite and aragonite phases undergo dissolution-reprecipitation, resulting in the predominance of calcite. In coal dust stabilization, calcite layers provide long-term consolidation, significantly improving resistance to wind and water erosion. Studies have demonstrated that calcite-rich matrices not only cement dust particles but also reduce porosity, thereby improving surface integrity and environmental durability (Du et al., 2024; Zhu et al., 2016).

Collectively, the sequential transformation from vaterite and aragonite to calcite underscores the dynamic nature of CaCO_3_ biomineralization in coal environments. The interplay between microbial metabolic pathways, mineral polymorphism, and coal dust particle surfaces determines the efficiency and sustainability of MICP-based dust suppression strategies.

### 2.2 Microbial taxa involved in MICP

The core of biomineralization technology lies in the activity of ureolytic bacteria, which catalyze the hydrolysis of urea, generating CO_3_^2−^ that reacts with calcium to form CaCO_3_. The following table outlines several bacterial species that have been studied in the context of MICP, particularly in coal-related environments (Table 2). Each species has distinct ecological sources, coal-type compatibility, catalytic pathways, and reported environmental or mechanical benefits. Notably, the table includes both well-established strains, such as *S. pasteurii*, and emerging candidates, such as *B. pseudofirmus* and *S. succinus* (Song et al., 2021). Their effectiveness varies based on environmental conditions such as pH, calcium availability, and moisture. Additionally, some bacteria have been engineered or used in combination to enhance overall performance (Yu et al., 2019a). Understanding the specific roles of each microorganism can guide the selection of strains for tailored applications in mining sites, coal storage facilities, and concrete structures exposed to coal dust. The inclusion of referenced studies ensures that these findings are backed by experimental evidence. These diverse microbial capabilities underscore the need to focus on well-characterized ureolytic species, particularly those whose physiological traits have been extensively evaluated in coal-associated MICP systems.

**Table 2:** Reported bacterial strains for MICP in coal dust control.

Producing microorganism	Isolated sources	Coal type, location	Reported effects	Mechanism of action	References
*Sporosarcina pasteurii*	Soil, alkaline environments	Bituminous (Shanxi, China)	Demonstrated high wind erosion resistance. Improved mechanical strength and dust stability of treated surfaces	Produces urease, catalyzing urea hydrolysis → carbonate formation → CaCO_3_ precipitation strengthens coal surfaces	Zhan et al. (2016), Li et al. (2024), Yu et al. (2019b), Wang H. et al. (2017)
*Paenibacillus enhance*	Soil	Bituminous (Ordos Basin, Inner Mongolia, China)	Forms CaCO_3_ layer that enhances dust binding capability	Urease activity promotes carbonate production and particle cementation. Produces extracellular polysaccharides and promotes mineralization	Li et al. (2024)
*Bacillus sphaericus*	Soil, wastewater environments	Lignite (Jharia Coalfield, India)	Reported potential in soil stabilization and biomineralization	Ureolytic pathway, binds calcium and CO_3_^2−^ to form CaCO_3_	Yu et al. (2018), Soon et al. (2013)
*Bacillus megaterium*	Soil	Coal dust (Inner Mongolia, China)	Improved dust wettability and wind resistance when combined with PVA	Enhances surface adhesion and carbonate binding, forming consolidated dust layers	Li et al. (2024), Bang et al. (2010), Jonkers et al. (2010)
Soil and rock samples		Bituminous (Shaanxi, China)	Potential in carbonate precipitation and dust stabilization	Participates in ureolysis and CaCO_3_ crystallization. Metabolizes urea to promote MICP in coal mine environments	Song et al. (2019)
*Bacillus pseudofirmus*	Alkaline soils	Anthracite (Datong Coalfield, China)	Used in self-healing concrete; relevant for biocement in coal matrix	Produces urease and EPS; facilitates MICP in high pH conditions. High tolerance to alkaline conditions. Urease production enables carbonate bonding of particles	Song et al. (2019)
*Bacillus sphaericus* and *Bacillus alkalinitrilicus*	Engineered microbial consortia	Anthracite (Shanxi, China)	Effective in crack sealing and biocementation applications	Synergistic urease activity; forms durable CaCO_3_ layers in porous media	Van Tittelboom et al. (2010), Wiktor and Jonkers (2011), Zhao et al. (2025), Siddique and Chahal (2011)
*Bacillus mucilaginosus*	Coal and soil environments	Bituminous (Ordos Basin, China)	Forms strong CaCO_3_ crusts that suppress dust effectively. CO_3_^2−^ and Ca^2+^ interact to create a stable CaCO_3_ crust that binds fine particles	Converts urea to carbonate, binds with calcium to form solidified structures. Cost-effective and biodegradable solution	Ryparová et al. (2021)
*Bacillus amyloliquefaciens*	Plant rhizosphere, coal areas	Lignite (Raniganj, India), Bituminous (Shanxi, China)	Exhibits urease activity and environmental tolerance; suitable for harsh mining conditions	Promotes rapid precipitation of CaCO_3_; suitable for biocementation of coal dust	Joshi et al. (2017)

MICP, microbially-induced calcium carbonate precipitation; EPS, extracellular polymeric substances; PVA, polyvinyl acetate.

*S. pasteurii*, *P. mucilaginosus*, *S. succinus*, and *B. mucilaginosus* were reported to be applied to bituminous coal to enhance dust stabilization and binding capacity. When applied to lignite coal, *B. sphaericus* and *B. amyloliquefaciens* were found to biomineralize the soil, with the latter demonstrating greater tolerance to harsh mining conditions. Furthermore, three *Bacillus* species (*B. pseudofirmus* applied as a monoculture, and *B. sphaericus* together with *B. alkalinitrilicus* applied as a consortium) were reported to exhibit good biocementation capacity of the coal matrix when tested in anthracite coal. In addition, the combined application of *B. megaterium* with polyvinyl acetate (PVA) showed promising results in improving resistance to wind-induced dust emissions. Overall, the abovementioned findings emphasize the potential of MICP in coal-related activities and indicate the need for continued, more in-depth research in this field.

The diversity of microbial species capable of inducing MICP in coal-associated environments highlights the expanding biotechnological potential of this process for sustainable mining operations and environmental remediation. Among the organisms investigated, *S. pasteurii* (formerly *B. pasteurii*) remains the most extensively studied due to its exceptionally high urease activity, strong CaCO_3_ precipitation capability, and ability to maintain metabolic function under a broad range of pH and temperature conditions (Jonkers et al., 2010; Zhang W. et al., 2023). Although *S. pasteurii* continues to serve as the benchmark organism for urease-driven biomineralization, increasing attention has been directed toward identifying additional microorganisms capable of tolerating the more challenging physicochemical conditions characteristic of coal mining environments.

Recent studies provided compelling evidence for the practical effectiveness of *S. pasteurii* as a microbial dust suppressant. Through laboratory simulations mimicking microenvironments of blast-dust fields, the authors demonstrated that *S. pasteurii* not only sustained robust growth but also maintained a stable urease activity of 7.78 mmol L ^− 1^ min ^− 1^ after 24 h in blast heap dust leachate (Chen et al., 2025). Wind and rain erosion experiments further confirmed that MICP-treated coal dust exhibited substantial improvements in resistance to mechanical disturbances, with wind erosion decreasing by 98.24, 86.99, 64.08, and 40.98% after four sequential impact events. Rain erosion resistance similarly increased by 75.55% after 35 min of simulated rainfall (Liu et al., 2023). These findings validate the feasibility of microbial dust suppressants as an environmentally friendly solution for mitigating particulate emissions in open-pit coal mine blast areas. Building on these laboratory and field evaluations, studies such as that by Li et al. (2024) have demonstrated that dust suppressants incorporating *S. pasteurii* XL-1 and glycerol can markedly improve surface mechanical properties and dust stability, reinforcing the promise of MICP as a practical solution for coal-mine dust mitigation (Du et al., 2024; Krajewska, 2009).

Building on these advances, recent research has expanded beyond *S. pasteurii* to explore new bacterial candidates that may perform more effectively under specific environmental constraints. For example, *Bacillus pseudofirmus* has demonstrated exceptional tolerance to the highly alkaline conditions typical of mining wastes, enabling sustained biomineralization under pH levels that inhibit many other species (Roux et al., 2026; Kliková et al., 2025; Šovljanski et al., 2021). Likewise, *B. mucilaginosus (Paenibacillus mucilaginosus)* exhibits a dual functional role: promoting CaCO_3_ precipitation while simultaneously enhancing soil aggregation and stability through the production of extracellular polysaccharides (Cheng and Cord-Ruwisch, 2012). The increasing recognition of such specialized bacteria underscores the value of broadening the microbial repertoire available for MICP, particularly for deployment in complex coal-derived environments where conditions may deviate substantially from standard laboratory settings.

Additional promising species include *S. succinus* and *B. amyloliquefaciens*, both of which show resilience in dust-prone and microbially diverse environments, making them suitable candidates for large-scale coal-site bioremediation (Yu et al., 2019b). *B. amyloliquefaciens* has been reported as an effective MICP-active strain due to its moderate urease activity and high production of EPS, which enhances bacterial adhesion to coal particle surfaces. These properties contribute to improved aggregation of coal dust particles and stabilization of calcium carbonate precipitates under variable environmental conditions. In parallel, the development of engineered microbial consortia, such as combinations of *B. sphaericus* and *Bacillus alkalinitrilicus,* has opened new opportunities to enhance biomineralization efficiency through synergistic metabolic interactions and complementary ureolytic pathways (Gupta et al., 2018). These advancements further illustrate the growing interest in designing tailored microbial systems optimized for field-relevant performance.

Among the alternative MICP-generating microorganisms, *B. sphaericus* has emerged as a particularly versatile species due to its ureolytic activity, spore-forming capability, and long-term survivability under harsh environmental conditions typical of coal-associated systems (Wang J. et al., 2017). Its biomineralization pathways facilitate CaCO_3_ precipitation that supports surface consolidation, pore sealing, and heavy-metal immobilization within overburden and coal tailings deposits (Seifan et al., 2017a). Recent work has shown that embedding *B. sphaericus* into carbon-rich carriers, including biochar and coal fly ash, enhances bacterial viability, nutrient distribution, and CaCO_3_ productivity (Seifan et al., 2017b). Although the incorporation of polymers may initially reduce mechanical strength, the long-term benefits include improved crack healing, decreased permeability, and sustained compressive strength recovery driven by dense calcite deposition (Seifan et al., 2018).

Environmental variables such as pH, oxygen availability, and calcium concentration play decisive roles in controlling MICP performance in coal-related settings. For example, studies have shown that adequate oxygen availability increases both urease activity and CaCO_3_ precipitation in *B. sphaericus* and *B. licheniformis*, while alkaline conditions (pH 9–12) facilitate the transformation of vaterite to stable calcite, conditions commonly encountered in alkaline mine drainage and coal fly ash environments (Shirakawa et al., 2015).

Innovative approaches have been proposed to support microbial activity in deeper or oxygen-limited strata. Oxygen-releasing compounds such as calcium peroxide have been successfully used to sustain microbial metabolism and promote CaCO_3_ precipitation in subsurface coal formations. Similarly, magnetic nanoparticle-assisted immobilization of *B. sphaericus* has been reported to accelerate calcite nucleation without altering crystal morphology, enabling more controlled and localized biomineralization. Long-term field studies confirm that *B. sphaericus* can generate compact, uniform calcite layers that enhance structural stability and reduce permeability, water absorption, and microbial degradation. When applied to coal gangue or cementitious composites, this biogenic calcite significantly improves durability.

Collectively, these findings position *B. sphaericus* as a strong candidate for MICP-driven remediation in coal-related environments. Its tolerance to high pH, salinity, and nutrient scarcity, combined with compatibility with carrier systems such as biochar, nanomaterials, and oxygen donors, underscores its potential for large-scale applications in eco-friendly construction, mine waste stabilization, and *in situ* carbon sequestration (Kumari and Sarkar, 2016). Nonetheless, further research is needed to better understand the interactions among microbial metabolism, mineral phase evolution, and the complex geochemistry of coal-derived matrices to optimize industrial-scale deployment.

#### 2.2.1 Urea-producing microorganisms

Microorganisms and microbially-mediated mineralization processes are active in almost every environment on Earth (Lin et al., 2021) and possibly in extraterrestrial systems (Su and Yang, 2021). In natural environments, the chemical precipitation of CaCO_3_ (Ca_2_^+^ + CO_3_^2−^ → CaCO_3_↓) is accompanied by biological processes, both of which often occur simultaneously or sequentially. Microbes from soils and aquatic environments often cause the precipitation of CaCO_3_ mineral phases both *in vivo* and in the laboratory (Lian et al., 2006). Therefore, microbial activity is considered an important player in the formation of carbonate precipitation and soil carbonate deposits (Anbu et al., 2016). Several studies have investigated microbial-mediated carbonate mineralization (see (Wright and Oren, 2005) and references therein), including mineralization by soil bacteria (Anbu et al., 2016; Cacchio et al., 2003; Wang et al., 2020). The important role of microorganisms that form urease in the natural environment in the formation of carbonate minerals is closely related to the diversity of their biochemical processes and, therefore, calls for a broader consideration of ways to implement MICP.

MICP can be accomplished through various microbial processes, such as urea hydrolysis, photosynthesis, sulfate reduction, nitrate reduction, or other microbial biochemical actions that increase the saturation state of carbonate (Chuo et al., 2020; Dhami, 2013; Van Mullem et al., 2020). MICP technology trends focus on soil improvement, crack sealing, self-healing concrete and heavy metal removal from water (Zheng et al., 2020). The bacteria used in these processes are mainly found in harsh environments with high alkalinity, nutrient deficiency and high shear. High viability and enzymatic activity of bacteria are critical factors for the success of MICP (Gorospe et al., 2013; Wang et al., 2016; Jiang et al., 2020). In some applications, such as self-healing concrete, where bacteria are incorporated into concrete during the mixing and pouring process, the use of alkali-tolerant bacterial spores is more favorable than the use of vegetative cells, as they can withstand harsh conditions (Pungrasmi et al., 2019). Understanding the biochemical basis of MICP directly affects its practical application, so strategies for selecting suitable bacterial strains are of particular importance for effective biomineralization in a specific environment.

The application of MICP in real environments can be achieved through biostimulation (Gowthaman et al., 2019a, 2019b) and bioaugmentation using either indigenous bacteria (strains obtained from local sites) (Vashisht et al., 2018; Arpajirakul et al., 2021; Omoregie et al., 2019a) or exogenous bacteria (non-indigenous strains) (Kim et al., 2016; Son et al., 2018; Wang et al., 2015; Lee et al., 2017; Iamchaturapatr et al., 2021; Yoon et al., 2017). The overall efficiency of *in situ* MICP treatments is largely governed by bacterial metabolic activity and by the complex interactions among ureolytic microorganisms, native microbial communities, and abiotic environmental factors. Consequently, identifying and selecting robust MICP-inducing strains represents a critical step toward successful field implementation. In this context, isolating and characterizing additional urease-producing bacteria, such as *Bacillus* (Fukano et al., 2015; Ahmed et al., 2007), *Lysinibacillus* (Mujah et al., 2017), *Pararhodobacter* (Zhu and Dittrich, 2016), and *Psychrobacillus* (Zhang et al., 2016), is essential for expanding the pool of bioresources capable of functioning under diverse climatic and environmental conditions. While broadening the catalog of ureolytic microorganisms facilitates the tailoring of strains to specific MICP applications, *S. pasteurii* remains one of the most widely utilized and extensively studied species due to its exceptional ureolytic activity and environmental tolerance. *S. pasteurii* can thrive in alkaline and nutrient-limited environments, making it a robust candidate for field applications in biocementation and dust control (Achal et al., 2009a). Its ability to form calcite through enzymatic hydrolysis of urea has been proven effective in stabilizing loose particles and repairing cracks in construction materials (Cheng and Cord-Ruwisch, 2012). Compared to traditional chemical methods of CaCO_3_ precipitation, the biological route through MICP offers substantial advantages, particularly in terms of reduced chemical input and lower energy demands. These efficiencies, combined with the minimal generation of secondary pollutants, make MICP a more practical and environmentally favorable alternative for large-scale applications. Furthermore, *S. pasteurii* can be incorporated into materials such as concrete in spore form, ensuring long-term survivability under harsh conditions (Omoregie et al., 2019b; Vashisht et al., 2018). Biogenic calcite formation occurs under ambient temperature and pressure, which eliminates the need for energy-intensive processing typical of chemical synthesis (DeJong et al., 2010). Additionally, microbial precipitation allows for controlled deposition of CaCO_3_, offering better integration with substrates such as soil, coal dust, or building materials. Consequently, MICP driven by *S. pasteurii* has emerged as a promising strategy for sustainable construction, mine site remediation, and dust suppression, particularly in arid and dust-prone environments.

#### 2.2.2 Other MICP-capable microorganisms

Beyond the widely studied *S. pasteurii* and *B. sphaericus*, a broad spectrum of additional bacterial strains has shown promise for MICP applications across diverse environments. For example, *Bacillus cereus*—an aerobic, Gram-positive urease producer has demonstrated bio-cementation capability, although its potential pathogenicity warrants caution during application (Gorospe et al., 2013; Rozenbaum et al., 2014). In mortar systems, *B. cereus* NS4 combined with 25% metakaolin has produced enhanced compressive strength and reduced permeability compared with control mixtures, highlighting its applicability in bio-enhanced construction materials (Li et al., 2017).

*Bacillus cohnii*, a non-ureolytic and alkaliphilic species, has been used in self-healing concrete via encapsulation in porous carriers such as expanded perlite and expanded clay. Utilizing calcium lactate rather than urea, this strain demonstrated significant crack-healing capacity within 28 days, with expanded perlite outperforming other carriers in water retention and oxygen diffusion (Zhang et al., 2017). Likewise, *B. pseudofirmus*, a spore-forming alkaliphile, has shown consistent though modest crack closure in autogenous healing mortars, and its performance improves when spores are injected alongside amino acids that stimulate germination (Sharma et al., 2017; Lors et al., 2017; Helmi et al., 2016).

Other species, such as *B. licheniformis*, have been shown to precipitate predominantly calcite with minor vaterite under optimal pH and temperature conditions, though certain calcium sources (e.g., calcium acetate) may inhibit precipitation due to associated pH reduction (Bai et al., 2017). Additional encapsulation innovations include the use of zeolite carriers for *Sporosarcina ureae*, which improve bacterial protection and enhance consistency despite slightly lower performance compared with *S. pasteurii* (Bhaskar et al., 2017). Meanwhile, *Paenibacillus mucilaginosus* demonstrates environmental value by facilitating CO_2_ uptake and dust agglomeration through MICP-driven biological processes.

Furthermore, nitrate-reducing bacteria such as *Pseudomonas aeruginosa* and *Diaphorobacter nitroreducens* have shown effective crack healing when immobilized in expanded clay or activated carbon, sealing cracks up to 400–500 μm and restoring water permeability by up to 85% within 28–56 days. Both calcite and aragonite were detected during mineralization, indicating the efficacy of nitrate reduction as a complementary pathway to ureolysis (Li et al., 2024). Additional insights from Bai et al. (2017) revealed distinctive biomineralization behavior of *P. aeruginosa* biofilms, while Lin et al. (2018) highlighted the sensitivity of nucleation and crystal morphology in *Desulfovibrio bizertensis*, particularly under phosphate-rich conditions that inhibit aragonite formation.

Taken together, these studies demonstrate that a wide variety of bacterial taxa possess MICP potential, each offering distinct metabolic pathways, environmental resilience, and carrier compatibility. While substantial progress has been made, continued research is necessary to refine strain selection, delivery strategies, and long-term performance under real-world coal mining conditions.

#### 2.2.3 Factors influencing MICP efficacy and comparative assessment of strain performance

A review of the literature indicates that the performance of MICP-capable strains is governed by a constellation of environmental factors, including pH, temperature, calcium concentration, and coal rank, with substantial variability in reported outcomes and several unresolved limitations affecting field deployment. Key findings that may serve as baseline values for comparative assessment are as follows:

- Baseline performance for ureolytic MICP: *S. pasteurii* ATCC 11859 achieved 84% efficacy in dust suppression at a wind speed of 10 m s^−1^ in coal environments following a single application (1 L m^−2^) (Zhou et al., 2023a).
- Baseline for synergistic CA acceleration: Mixed-strain systems (*B. sphaericus* + *B. mucilaginosus*) achieved a 1.89-fold enhancement in CaCO_3_ precipitation relative to *B. sphaericus* monoculture (Hu et al., 2020).
- Baseline for the lowest documented MICP temperature: Psychrotolerant *Rhodococcus* strains enable MICP operation at temperatures as low as 5 °C (Shen et al., 2025).

##### 2.2.3.1 pH-dependent performance

pH represents the primary environmental determinant of MICP efficacy; therefore, coal type must be considered when selecting the most suitable strains for a given MICP strategy. Coal dust exhibits variable pH buffering capacity: bituminous coals (pH ~ 7–8), lignite (pH ~ 5–6), and anthracite (pH ~ 6–7). In addition to coal type, the MICP pathway utilized by microorganisms differs in its ability to modify environmental pH. For example, ureolytic bacteria typically reach optimal performance under neutral to slightly alkaline conditions (pH 6.5–8.0) while simultaneously self-regulating pH toward 9 through urea hydrolysis (Dong Y. et al., 2023). By contrast, in acidic coal environments (e.g., lignite with pH ~ 5–6), strains exploiting CA activity, which accelerates CO_2_ hydration up to 100-fold, can markedly increase pH via the H_2_CO_3_ → HCO_3_^−^ → CO_3_^2−^ cascade, thereby effectively buffering the acidic coal dust microenvironment (Zhou et al., 2025). In strongly alkaline environments (such as concrete with pH 11–13), the use of spore-forming ureolytic bacteria (e.g., *B. sphaericus* LMG 22257) has been reported to facilitate the attainment of optimal MICP efficacy (Wang J. et al., 2017).

Overall, the initial coal-associated environmental pH establishes the baseline for enzyme kinetics, followed by pH self-regulation driven primarily by urea hydrolysis. However, pH fluctuations exceeding one unit have been shown to reduce peak urease activity by 20–40%, thereby affecting both precipitation rate and total CaCO_3_ yield (Dong Y. et al., 2023). Consequently, in highly acidic environments, particularly lignite, CA-exploiting bacterial strains are preferable; in neutral to slightly alkaline environments, such as bituminous coal and anthracite, ureolytic bacteria are more suitable; and in strongly alkaline environments, including concrete, spore-forming bacteria are recommended.

##### 2.2.3.2 Temperature-driven performance

Ureolytic strains typically follow mesophilic enzyme kinetics, with an optimal temperature of approximately 30 °C. An investigation of urease activity in *S. pasteurii’s* across a temperature range of 20–40 °C demonstrated a 45.9% reduction in activity at 20 °C compared with the optimal urease activity peak observed at 30–35 °C (Dong Y. et al., 2023). In addition, another study examining the effects of temperature on urease activity and the rate of carbon mineralization in *S. pasteurii* ATCC11859 reported that optimal values for both parameters were attained at 37 °C (Li et al., 2022). By contrast, analysis of urease activity in psychrotolerant *Rhodococcus* sp. L6 and *Rhodococcus* sp. L8 indicated that a decrease in temperature from the optimum to 20 °C did not affect urease activity, although the time required for mineralization was extended from 24 to 36 h (Shen et al., 2025).

Considering that real-scale MICP applications in coal mining typically involve spray application onto exposed surfaces, surface temperature represents an additional limiting factor. During summer, solar heating during daylight hours generates pronounced local temperature gradients, with surface temperatures reaching 40–50 °C under direct sunlight and remaining at 5–15 °C in shaded areas; during autumn and spring, surface temperatures of approximately 10 °C are common in temperate mining regions. In cold-climate coal mining, low positive temperatures prevail throughout the year. Consequently, effective implementation of MICP requires the combined application of strains active across a wide temperature range. Accordingly, these scenarios necessitate systematic strain substitution, with strains active at low temperatures being more suitable for permanent deployments, whereas strains active at higher positive temperatures (25–37 °C) are more appropriate for seasonally optimized conditions, particularly during spring and summer.

##### 2.2.3.3 Coal rank-dependent performance

Although coal type, and consequently coal rank, is expected to influence MICP efficiency owing to differences in porosity, carbon content, and dust particle size, no published studies have directly compared coal rank-dependent urease activity in MICP-active bacterial strains. Despite the sufficient characterization of individual components of MICP-based dust suppression systems, their interactions across different coal types remain underexplored; therefore, this research gap requires further investigation. The most frequently reported high-performing MICP strain is *S. pasteurii* (Zhou et al., 2025), with emerging potential also reported for *B. megaterium* and *B. subtilis* strains. Coal structural parameters vary markedly across ranks (lignite specific surface area (SSA):2.30 m^2^ g^−1^; bituminous coal: 3.17 m^2^ g^−1^; anthracite: 1.37 m^2^ g^−1^) (Shen et al., 2025); however, no studies have systematically related these differences to MICP efficiency. A limited number of studies have indicated variations in CaCO_3_ yield depending on coal rank, resulting in the following lignite:bituminous:anthracite ratios: for urease-producing microbial system, 1.25:1:1.17 (Zhao et al., 2024), for *S. succinus* J3, 1.13:1:1.07 (Song et al., 2019); and for *B. pasteurii* co-cultured with *B. mucilaginosus* 1.22:1:1.13 (Zhou et al., 2025).

## 3 Role of microbially-induced calcium carbonate precipitation in coal dust suppression

Currently, microbial dust suppressants are attracting significant research interest due to their effectiveness and environmentally friendly nature in controlling dust. A crucial aspect of their functionality lies in the adsorption of urease-producing bacteria onto coal dust, which facilitates the release of urease and enhances dust compaction. Despite the limited amount of research on this topic, a study by Zhang et al. (2022) aimed to shed light on the dust suppression modes of action employed by microbial dust suppressants. To achieve this, they investigated the adsorption behaviors and interaction processes between coal dust and the urease-producing bacterium *Bacillus X4* (*B.* X4) (Zhang et al., 2022). Furthermore, they established a connection between the effectiveness of adsorption and dust suppression. Their results indicated that *B. X4* bacteria exhibited the greatest adsorption capacity for coal particles in the 40–80 mesh size range (40.71 mg g^−1^), demonstrating a 1.61-fold higher affinity compared to 120–200 mesh coal dust. Scanning electron microscopy (SEM) and adsorption isotherms showed that a large number of bacteria could be adsorbed on the surface of coal dust, and the bacteria attached through monolayer adsorption. Fourier transform infrared spectroscopy (FTIR) analysis showed that the amide group on the surface of bacteria was the leading active group for bacterial adsorption on coal dust. The results showed that *Bacillus X4* bacteria improved the water wettability of the coal dust surface. It was also known that the large particle size of coal dust resulted in a higher content of CaCO_3_ formed after spraying the microbial dust suppressant. The microbial dust suppressant showed the best results when sprayed on the coal dust with a mesh size of 40–80, which was consistent with the adsorption analysis results, indicating that the amount of microorganisms adsorbed on coal dust was positively correlated with the dust suppression effect (Zhang et al., 2022).

According to the research of Fang et al. (2020) and Li S. et al. (2021), various dust prevention and control methods have been adopted to solve the coal dust pollution problem, including the use of dust covering agents, water spray dust removal (Fang et al., 2020), chemical dust suppression (Li S. et al., 2021) and surface curing. In their recent review, Seifan and Berenjian (2019) pointed out that among the applied surface curing methods, MICP technology has attracted wide attention from researchers in recent years due to their low energy consumption, high efficiency and low environmental impact. Also, in the research of Rahman et al. (2020), Song et al. (2019), Shi et al. (2022) showed that microbial dust suppressants use CaCO_3_ formed during the growth of urease-producing bacteria as a cementing agent to fix coal dust. Urease-producing bacteria have high CaCO_3_ production efficiency, and are easy to control. Therefore, various dust suppressants based on MICP technology were developed and their suitability in the field of dust suppression was tested (Wu et al., 2020; Fan et al., 2020; Zhu et al., 2021), showing that the wind- and water-erosion resistance of coal dust-fixed microbial dust suppressant was significantly higher than that of coal dust-fixed chemical dust suppressant (Yi et al., 2021). For microbial dust suppressants, the ability of microorganisms to adsorb on coal dust and form a smooth coating layer plays a key role, since this affects the secretion of urease and biomineralization, thereby affecting the efficiency of dust suppression (Shi et al., 2022; Nalini and Prakasham, 2022).

MICP-based dust suppressants have emerged as a new and effective method for dust suppression in recent years; however, their effectiveness is strongly influenced by environmental conditions, and its action mechanism is complex. It was found that the dust suppression efficiency was highest when the ratio of bacterial solution to cementing mortar was 2:1 and the 15-day wind erosion rate was 0.68%. In the early stage, the nutrients in the bacterial solution bound the coal mass, suppressing dust release. However, the dominant factor for the continuous performance of subsequent dust suppression was the precipitation and adhesion of CaCO_3_ generated by biomineralization. As the reaction progressed, the crystal form of CaCO_3_ gradually changed from the original vaterite to calcite. When the proportion of bacterial solution was high, it promoted this transformation and stabilized the cementation. However, when the proportion of bacterial solution was low, the transformation time of vaterite to calcite increased and the proportion of calcite decreased.

**Figure 3:**
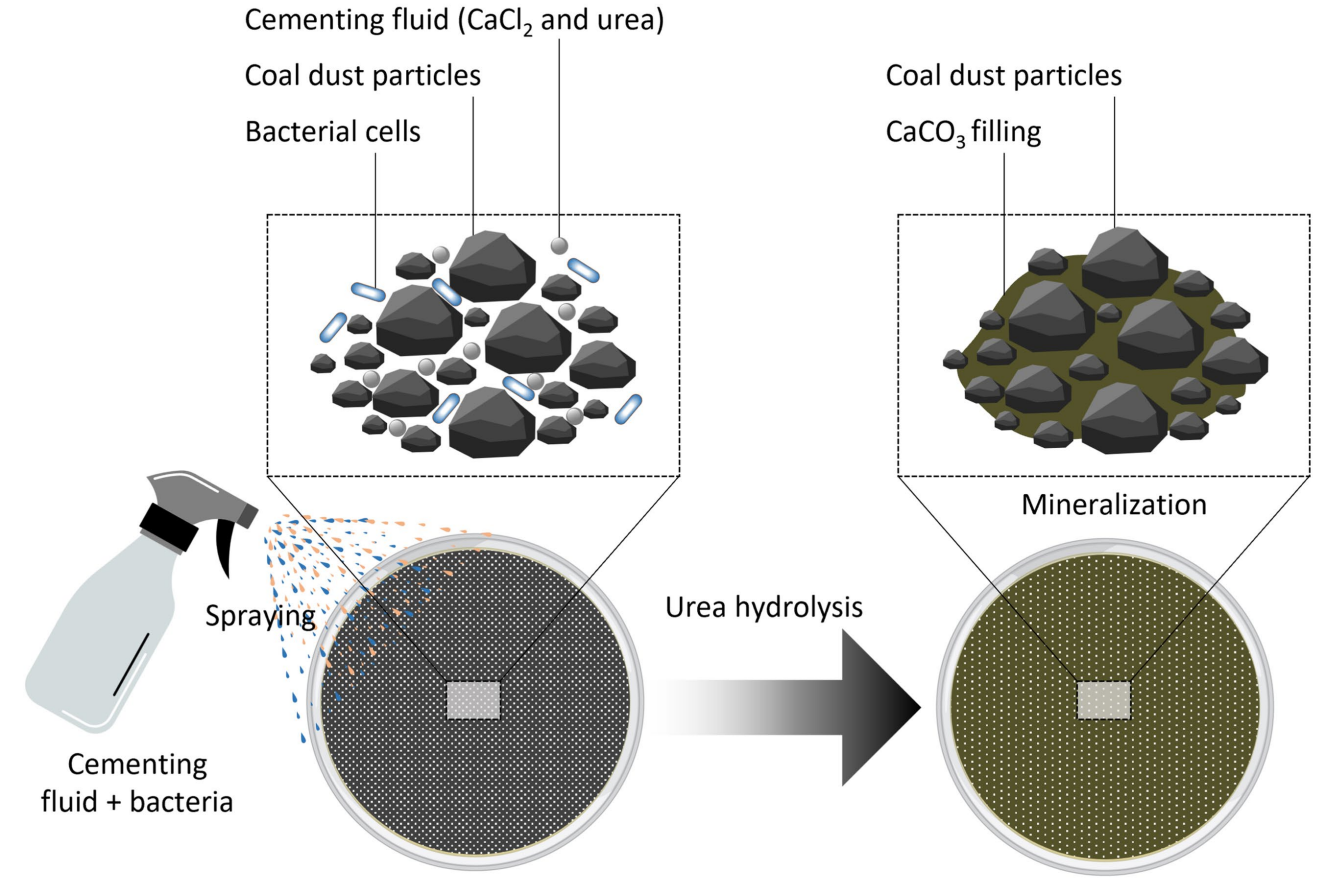
MICP-assisted coal dust suppression.

The whole procedure for dust suppressant application is illustrated in Figure 3. Many urease-producing bacteria have significant tolerance to extreme environments, including acidic, alkaline, and saline conditions, while sustaining strong biological activity at temperatures between 15 and 37 °C. Moreover, these bacteria excrete significant quantities of urease via their metabolic activities. The introduction of cementing fluid (urea and a soluble calcium source) facilitates the reaction between CO_3_^2−^ and Ca^2+^, resulting in the formation of CaCO_3_ precipitate (Chen et al., 2025). The significant deposition of CaCO_3_ on microbial surfaces enhances interaction between CO_3_^2−^ accumulations on adjacent particles. This interaction gradually reduces the interstitial spaces between dust particles, resulting in the development of a consolidating CaCO_3_ coating on the surfaces of the particles.

The cementation effect of MICP technology is influenced by various factors, and changes in these factors often lead to different effects and actions. The study by Bu et al. (2018) demonstrated that Tris significantly enhanced the unconfined compressive strength of the specimen during MICP treatment, approaching the effect of a 20–25% cement treatment. Also, Peng et al. (2022) found that different precipitation environments and calcium sources controlled the production of CaCO_3_. The production of CaCl_2_ in freshwater was 2.0% higher than that in seawater, while the production of calcium acetate (Ca(C_2_H_3_O_2_)_2_) and calcium nitrate (Ca(NO_3_)_2_) increased by 7.5 and 25.0%, respectively, in the same environment.

The strength of biocemented specimens was significantly improved when magnesium ions were added to the cementing mortar. Wang J. et al. (2024) found that the hardness of the soil consolidation layer increased with the increase of molasses concentration, and the wind erosion rate decreased by 93.64% under 1% molasses treatment. In another study, Liu et al. (2023) focused on the effects of different EPS on the dust suppression performance of microbial dust suppressants. The results showed that with the addition of a quantitative amount of EPS, the wind erosion resistance of the consolidated specimen increased by 189.0% compared with the traditional treatment. From this, it could be seen that improving the composition and concentration of dust suppressants could enhance the binding effect of MICP. However, few studies have investigated the effects of the proportion of bacterial solution and cementing mortar on the dust consolidation mechanism. Beyond these material and environmental influences, the biochemical pathways underlying MICP also play a critical role in determining the form and rate of CaCO_3_ precipitation.

### 3.1 Biochemical pathways

Apart from ureolysis, various microbial metabolic pathways can induce CaCO_3_ precipitation (Xu et al., 2020). These include:

- Denitrification: facultative anaerobic bacteria reduce nitrate (NO_3_^−^) to nitrogen gas (N_2_), generating alkalinity that promotes the precipitation of carbonate. This pathway is advantageous in oxygen-limited environments.
- Sulfate reduction: sulfate-reducing bacteria reduce sulfate (SO_4_^2−^) to hydrogen sulfide (H_2_S), increasing pH and carbonate ion availability. This process is effective in anoxic, organic-rich environments like sediments.
- Photosynthesis: photosynthetic organisms like cyanobacteria uptake CO_2_ and increase pH through photosynthetic carbon fixation, leading to supersaturation of carbonate and Ca^2+^.

Each of these pathways modifies the surrounding microenvironment to favor CaCO_3_ precipitation, though their rates and effectiveness vary based on microbial activity and environmental conditions.

### 3.2 Environmental factors affecting precipitation efficiency

Several physicochemical factors influence the efficiency of CaCO_3_ precipitation via microbial activity:

- pH: a higher pH (typically 8–9.5) facilitates the conversion of CO_2_ to CO_3_^2−^, promoting supersaturation and nucleation of CaCO_3_. Alkalinity is often a limiting factor in non-ureolytic pathways.
- Ca^2+^ concentration: maintaining sufficient levels of free Ca^2+^ is crucial for adequate precipitation. The source of calcium can vary (e.g., calcium chloride, calcium acetate).
- Temperature: optimal bacterial activity for MICP usually occurs between 25 °C and 37 °C. Elevated temperatures may denature enzymes, while low temperatures slow microbial metabolism.
- Salinity and ionic strength: can impact microbial growth, enzyme activity, and the solubility of CaCO_3_. High-ionic-strength environments, such as seawater, may require salt-tolerant strains.
- Urea concentration: while necessary for ureolysis, excessive urea can lead to NH_3_ toxicity or osmotic stress, impacting cell viability.

Understanding and optimizing these environmental parameters is essential for scaling MICP in practical applications such as geotechnical engineering, carbon sequestration, and environmental remediation. These optimized environmental conditions are essential for translating laboratory-scale MICP processes into effective field applications, particularly in environmentally sensitive settings.

**Figure 4:**
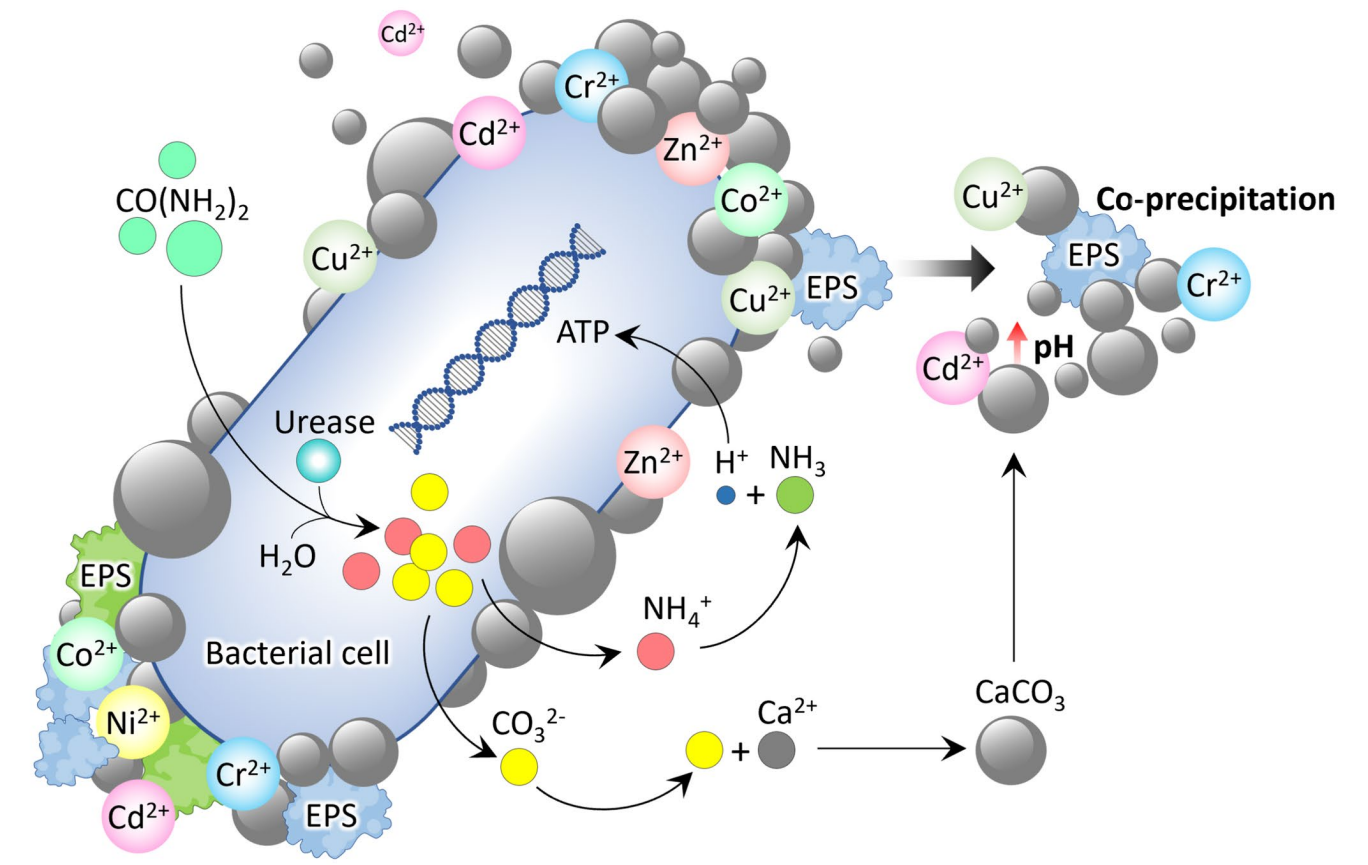
MICP facilitated by bacterial actions to precipitate diverse heavy metals.

The data presented in Table 3 emphasizes the growing importance of MICP in mitigating the environmental impacts of coal-related operations. Additionally, the MICP process has shown the ability to immobilize heavy metals by co-precipitating them with carbonate minerals, reducing their mobility and ecological risk. The process typically involves adding urea and a calcium source to a contaminated site, where the urease enzyme hydrolyzes the urea to produce CO_3_^2−^ and increase the environmental pH. The CO_3_^2−^ then react with both added calcium and the heavy metal ions (Cd, Cr, Cu, Pb, Ni, Zn) present to form insoluble metal carbonates, effectively trapping and converting the toxic, mobile heavy metals into stable, less bioavailable solid forms (Figure 4). Furthermore, bacterial surfaces are vital for calcium and heavy metal precipitation because their EPS (proteins, polysaccharides, nucleic acids, humic acid) acts as a nucleation site for mineral formation, while their metabolic activity increases local carbonate ion concentration and pH, shifting the solution toward supersaturation and mineral precipitation (Al Qabany et al., 2012). This environmentally friendly method holds great potential for remediation, although its efficiency can be influenced by heavy metal concentration, pH, and bacterial tolerance.

**Table 3:** The main aspects of MICP application in the coal sector.

Aspects	Details
Main pollutants in coal industry	Coal dust, heavy metals (e.g., Pb, Cd, Zn), AMD
Bioremediation approach	MICP
Key bacterial species	*S. pasteurii*, *B. megaterium*, *B. sphaericus*, *P. mucilaginosus*
Primary enzyme involved	Urease (catalyzes urea → NH_4_^+^ + carbonate)
Mechanism of action	Urease-producing bacteria hydrolyze urea, increasing pH and generating CO_3_^2−^. These ions react with Ca^2+^ to form insoluble CaCO_3_. The CaCO_3_ precipitate binds fine dust particles, fills surface pores, and forms a hardened crust that stabilizes the material and prevents erosion
Coal types treated	Bituminous, lignite, anthracite
Environmental benefits	Dust suppression, heavy metal immobilization, AMD pH neutralization, soil stabilization
Delivery methods	Spraying bacterial solution with urea and calcium on coal surfaces or tailings
Challenges	Formation of ammonia as a by-product, need for nutrient optimization, sensitivity to moisture, pH, and temperature
Outlook	High potential for eco-friendly remediation and sustainable mine site management

MICP, microbially-induced carbonate precipitation; AMD, acid mine drainage.

Another important application is the neutralization of acid mine drainage (AMD), where microbial carbonate production increases local pH and facilitates sulfate and metal precipitation. Despite these benefits, several challenges remain, including the release of ammonia during ureolysis and the need for optimal moisture, temperature, and nutrient conditions to support microbial activity. Nonetheless, with ongoing advances in microbial engineering, nutrient optimization, and delivery methods, the future of MICP in coal-related environmental remediation is highly promising. As regulatory pressure for greener technologies grows, MICP stands out as a viable and scalable solution for coal companies seeking sustainable site management strategies. The integration of this biotechnological approach into modern mining practices could significantly reduce ecological damage and improve post-mining land recovery.

With the development of science and technology, microbial dust suppressants based on MICP technology have also attracted the research interests of scientists in the occupational safety industry.

In studies by Yinnan and Ruxiang (2023), it was shown that during the extraction, processing and transportation of coal, the dust concentration in the mine area reached 3,000 mg m^−3^, exceeding the norm by 86.5%. High dust concentration, if not treated and discharged, will increase the cost of coal producers and affect the production process of enterprises (Yinnan and Ruxiang, 2023). At the same time, coal dust, a potentially harmful element generated during the mining process, can pose a serious threat to the health of miners when released into the air (Gopinathan et al., 2024; Tong et al., 2019). In particular, the accumulation and emission of coal dust can also easily lead to dust explosions (Yang et al., 2024), posing a significant potential danger to production safety in mining areas (Zheng et al., 2009). Therefore, the research, development and improvement of dust control technologies in high-dust mine areas are of great significance to the safe production and green development of coal mines.

Wang H. et al. (2017) divided the commonly used dust suppression methods into two main categories: physical methods and chemical methods. Physical spraying requires a large amount of water resources and needs to be sprayed repeatedly to maintain the dust suppression effect (Wang et al., 2023); deploying dust nets can be effective in preventing and controlling dust, but their installation and dismantling are labor-intensive and wasteful, and there is a potential danger of microplastic pollution (Chen et al., 2021). Chemical dust suppressants can be divided into two types, wetting-type and binding-type, based on their action principles (Liao et al., 2018). Wetting-type dust suppressants wet the dust to form a liquid film on the surface of the dust, trapping the dust and preventing it from being released into the air due to external interference (Gowthaman et al., 2021); binding-type dust suppressants, obtained through molecular modification using polyacrylamide and other polymer compounds as raw materials, can effectively control dust diffusion. However, Dong H. et al. (2023) described that the use of chemical dust suppressants, led to the gradual emergence of secondary pollution from chemical reagents, and the issues of corrosivity, toxicity and degradation complexity also became a subject of discussion in the academic community. These limitations have prompted researchers to seek safer, more sustainable alternatives capable of delivering long-term dust suppression without adverse environmental impacts. Therefore, the study of environmentally friendly and effective dust suppression methods is a new direction in the development of dust suppression technologies.

To address the above problems, researchers have investigated the ability of bacterial spore to induce the precipitation of CaCO_3_ for self-healing cement. Microbial bacterial powder is a living bacterial agent obtained by treating target microorganisms after industrial production and expanding the use of porous carriers as adsorbents, which protects bacterial strains from adverse environmental influences and maintains their activity and function. Some researchers, such as Xu et al. (2021) and Rahmaninezhad et al. (2024) prepared self-healing cement and achieved good crack repair by inducing bacteria to produce spores through thermal excitation; studies on the mineralization caused by bacterial spore germination show that the spore germination rate is a key factor affecting the degree of biomineralization (Huang et al., 2023). Meanwhile, with the advantage that microbial bacterial powder requires low activation conditions, it has been widely applied in the fields of agriculture and animal husbandry (Zalila-Kolsi et al., 2023). However, little research has been conducted in the field of dust suppression. From the consideration of environmental adaptability and convenient operation, if the commercial bacterial powder is used in the microbial dust suppression process, the strain is protected by the porous adsorbent material to maintain viability and stable function; on the other hand, the direct use of tap water lyses the bacteria, which greatly shortens the operation of activating the culture of internal strains, and the operation is straightforward and convenient, saving much time. On the one hand, in their experiments, Guo et al. (2025) developed a microbial dust suppressant by mixing commercial microbial powder with tap water. They studied the effectiveness of this microbial dust suppressant in reducing dust in coal mine dumps. The findings demonstrated that a commercial *B. amyloliquefaciens* had excellent dust suppression performance, forming a solidified layer over 1 cm thick in the surface layer of the discharge field.

MICP has shown considerable promise as a dust suppressant, particularly in mining and industrial environments. By biologically binding fine particles into a hardened crust, MICP effectively reduces airborne dust, improving air quality and worker safety. Field and laboratory studies have demonstrated its reliability across a range of environmental conditions, including arid and semi-arid regions. However, despite its potential, several challenges remain - most notably sensitivity to temperature, pH, moisture variation, and the release of ammonia as a by-product. These limitations highlight the need for targeted innovation to optimize bacterial performance and system efficiency under realistic field settings.

## 4 Challenges and future trends

Currently, MICP is emerging as a promising strategy for soil improvement in coal mining environments, AMD treatment, and mine tailings remediation. However, in addition to biological factors (e.g., environmental pH, temperature, coal rank, and strain-specific urease- or carbonic anhydrase (CA)-producing activity), several economic factors constrain process scalability. The principal cost drivers of MICP are nutrient sources required for bacterial cultivation and calcium sources. Multiple techno-economic analyses of MICP scalability have identified bacterial cultivation as the most expensive process component. In particular, *S. pasteurii*, the most widely used MICP-active strain, is typically cultured in complex media containing peptone or yeast extract (Williams et al., 2016; Cuzman et al., 2015), yet yields only moderate biomass (OD_600_ < 5) (Lapierre et al., 2020). This limitation renders industrial-scale cultivation economically unfavorable, as higher cell densities are required to reduce per-unit production costs and improve cost-effectiveness metrics (EUR L^−1^ OD_600_^−1^).

Consequently, substantial efforts have been directed toward the development of optimized culture media capable of achieving higher cell densities (microbial biomass yield) to improve cost-effectiveness (Maleki-Kakelar et al., 2022a; Achal et al., 2009b; Kahani et al., 2020). For example, cultivation of *S. pasteurii* DSM33 on CaSo medium supplemented with additional glucose, phosphate, and trace elements resulted in an approximately 400% increase in OD_600_, while increasing medium cost by only 4.3% (Lapierre et al., 2020). In parallel, replacement of yeast extract with meat extract and sodium acetate led to a 75% reduction in growth retardation of *S. pasteurii* ATCC 6453 compared with yeast extract, without compromising bacterial growth, urea hydrolysis, or calcium carbonate yield (Williams et al., 2016). Another effective approach for cost reduction involves the utilization of waste-derived media, which have demonstrated substantial decreases in production costs while maintaining comparable urease activity and MICP performance. Several waste streams have been validated at laboratory and pilot scales, including kitchen waste, chicken manure wastewater, tofu wastewater, whey and other dairy wastes, lactose mother liquor, corn steep liquor, food-grade yeast extract, and sugarcane molasses or vinasse (Yan et al., 2025).

In particular, wastes from the dairy industry (buttermilk, lactose mother liquor, whey, and cleaning-in-place wastewater) and brewery industry (spent yeasts) used as alternative nutrient sources for *S. pasteurii* DSM33 cultivation resulted in a 200-fold reduction in medium cost (Cuzman et al., 2015). Substitution of the nitrogen source with corn steep liquor for biocementation using *S. pasteurii* PTCC 1645 reduced medium cost from 7.5 to 2.05 USD L^−1^ (Maleki-Kakelar et al., 2022b). Similarly, replacement of soybean peptone with corn steep liquor during cultivation of *S. pasteurii* ATCC 11859 reduced medium cost by 50.5%, while simultaneously increasing urease activity by 24.2% (Chen et al., 2022). Overall, investigations into cost reduction strategies for MICP have already yielded encouraging results that contribute to improved process scalability; nevertheless, further research in this area remains necessary.

Looking forward, the evolution of MICP technology is expected to center on the development of resilient microbial strains capable of withstanding extreme and fluctuating environmental conditions. Advances in synthetic biology and metabolic engineering may allow the customization of bacterial systems tailored for specific deployment environments. Researchers are also exploring alternative nutrient sources and eco-compatible calcium carriers to enhance the environmental profile and efficiency of MICP-based approaches (De Muynck et al., 2010). Moreover, the integration of smart delivery systems, such as responsive spraying processes or encapsulated bacterial spores, could significantly improve field applicability and durability (van Paassen et al., 2010). Ultimately, the goal is to establish MICP as a cost-effective and versatile biotechnological tool extending beyond dust control to encompass wider environmental remediation applications.

Future deployment of MICP in coal-related settings requires overcoming current technical, economic, and ecological barriers. One key direction involves genetic enhancement of bacterial strains to tolerate high temperatures, alkalinity, and low moisture levels typical of mining environments. Modern molecular tools, including CRISPR/Cas and synthetic biology platforms, can be harnessed to strengthen traits such as urease activity, sporulation, and stress resistance, thereby improving microbial reliability under field conditions (Zhang K. et al., 2023).

Exploration of alternative calcium and nitrogen sources is gaining attention. Traditional reagents like calcium chloride and urea are costly and may generate undesirable byproducts, such as ammonia. Utilizing industrial byproducts, including blast furnace slag, seawater, or organic waste offers a more sustainable and economically feasible solution (Yu et al., 2022).

Maintaining bacterial viability during storage and after field application remains a crucial challenge. Advanced carriers, including biochar, expanded perlite, and hydrogel beads, have shown promise in protecting microbial cells from desiccation and ultraviolet (UV) damage while enabling controlled release of nutrients and cells. Automation technologies, such as drone-assisted spraying and robotic delivery systems, can further enhance scalability and precision across large coal yards and open-pit mines. In addition, hybrid formulations combining MICP with polymers, plant fibers, or nanomaterials have demonstrated synergistic improvements in adhesion, surface consolidation, and mechanical stability. For instance, integrating polyvinyl acetate (PVA) or geopolymer coatings with microbial treatments has been shown to significantly enhance the structural integrity of treated substrates (Geng et al., 2023).

Artificial intelligence and computational modeling offer transformative tools to predict MICP performance under variable field conditions, optimize strain selection, and minimize experimental uncertainty (Bao et al., 2024). Data-driven approaches can therefore accelerate the transition from laboratory-scale studies to full-scale implementation.

A promising avenue lies in integrating MICP with ecological stabilization measures such as vegetative covers. Microbial precipitation of CaCO_3_ consolidates loose particulates into a hardened crust, while vegetation increases surface roughness, traps dust particles, and maintains soil cohesion. This synergy enhances hydraulic stability, moisture retention, and microbial activity, enabling sustained carbonate formation. Vegetation further reinforces the crust through root networks and organic matter deposition, which promote natural self-healing of minor erosions. In arid regions, microbial mineralization has been shown to effectively bind sandy substrates, providing erosion resistance with minimal energy and carbon input, an approach that aligns with eco-restoration objectives (Zhevtun et al., 2024). Moreover, ecological covers reduce wind velocity near the surface and create favorable microclimates that promote long-term biological consolidation. Studies have demonstrated that microbial crusts supplemented with vegetation or fibrous reinforcement exhibit greater durability and reduced permeability (Taharia et al., 2024). Such dual-layer systems could be particularly beneficial for slope stabilization near coal waste piles and along dust-prone haul roads. Together, MICP provides rapid surface strengthening, while vegetation ensures ongoing resilience, forming an integrated and sustainable pathway for dust mitigation in mining environments.

Finally, supportive regulatory and commercialization frameworks will be essential for the safe and widespread implementation of MICP technologies. Clear biosafety standards, environmental assessments, and collaborative efforts among academia, industry, and government agencies are crucial for scaling and approval processes. Collectively, these advances point toward a more adaptive, efficient, and environmentally responsible use of MICP in the coal industry. With sustained interdisciplinary collaboration, MICP may evolve into a cornerstone technology for environmentally sound land management and industrial resilience.

## 5 Conclusion

Microbially-induced carbonate precipitation (MICP) represents a biologically driven mineralization process with broad applicability across environmental and engineering fields, particularly for the mitigation of particulate emissions in coal mining operations. This review has outlined the biological basis of MICP, with an emphasis on ureolytic processes and carbonate precipitation mediated by specialized bacterial strains. Laboratory- and pilot-scale investigations have confirmed the capacity of MICP to reduce dust emissions, immobilize heavy metals, and enhance soil and surface stability. Furthermore, techno-economic analysis has identified key cost drivers and demonstrated effective approaches for improving process cost-effectiveness. However, despite significant advances in cost optimization and in defining optimal operating conditions for specific MICP-active strains, several limiting factors remain insufficiently explored, including the development of coal rank-specific strategies and co-culturing approaches to overcome temperature-dependent declines in MICP efficiency. Therefore, future research should address these concerns and prioritize microbial strain improvement, intelligent delivery systems, and adaptive control strategies for field-scale applications. Continued innovation and interdisciplinary integration may ultimately establish MICP as a reliable, low-impact biotechnological approach for particulate mitigation and broader environmental rehabilitation efforts.
